# Hyperglycemia on Admission Predicts Acute Kidney Failure and Renal Functional Recovery among Inpatients

**DOI:** 10.3390/jcm11010054

**Published:** 2021-12-23

**Authors:** Yuri Gorelik, Natalie Bloch-Isenberg, Siwar Hashoul, Samuel N. Heyman, Mogher Khamaisi

**Affiliations:** 1 Department of Medicine D, Rambam Health Care Campus, Haifa 3109601, Israel; yurigorelik@gmail.com (Y.G.); Bloch.natalie@gmail.com (N.B.-I.); m_khamaisi@rambam.health.gov.il (M.K.); 2 Department of Medicine A, Ruth & Bruce Rappaport Faculty of Medicine, Technion-IIT, Haifa 3109601, Israel; h.siwar@gmail.com; 3 Department of Medicine A, Rambam Health Care Campus, Haifa 3109601, Israel; 4 Department of Medicine, Hadassah Hebrew University Hospital, Mt. Scopus, Jerusalem 91240, Israel

**Keywords:** hyperglycemia, acute kidney injury, kidney functional recovery, inpatients

## Abstract

Background: Hyperglycemia is associated with adverse outcomes in hospitalized patients. We aimed to assess the impact of glucose levels upon admission on the subsequent deterioration or improvement of kidney function in inpatients with a focus on diabetes or reduced baseline kidney function as possible modifiers of this effect. Methods: Running a retrospective cohort analysis, we compared patients with normal vs. high glucose levels upon admission. We applied multivariable logistic regression models to study the association between baseline glucose levels with subsequent renal and clinical outcomes. Interaction terms were used to study a possible modifier effect of diabetes. Results: Among 95,556 inpatients (52% males, mean age 61 years), 15,675 (16.5%) had plasma glucose higher than 180 mg/dL, and 72% of them were diabetics. Patients with higher glucose at presentation were older, with a higher proportion of co-morbid conditions. Rates of acute kidney injury (AKI), acute kidney functional recovery (AKR), and mortality were proportional to reduced renal function. AKI, AKR, and mortality were almost doubled in patients with high baseline glucose upon admission. Multivariable analysis with interaction terms demonstrated an increasing adjusted probability of all events as glucose increased, yet this association was observed principally in non-diabetic patients. Conclusions: Hyperglycemia is associated with AKI, AKR, and mortality in non-diabetic inpatients in proportion to the severity of their acute illness. This association diminishes in diabetic patients, suggesting a possible impact of treatable and easily reversible renal derangement in this population.

## 1. Introduction

The risk of acute kidney injury (AKI) among patients hospitalized with an acute illness is substantial and leads to a longer hospitalization course, morbidity and mortality [1]. Diabetes is a well-recognized risk factor for the development of AKI in various clinical setups. For instance, it was found to independently predict contrast nephropathy among patients with advanced renal impairment undergoing computerized tomography (CT) in a large propensity matched study [2]. Likewise, it was found to double the risk of AKI following coronary interventions for any given baseline renal function in a host of 3695 patients undergoing cardiac catheterization [3]. Accordingly, diabetes has been defined as a principal factor in a scoring system predicting the risk of AKI and the need for dialysis in this clinical setup [4]. Hyperglycemia per se might play a role under such circumstances through a variety of mechanisms, including renal hypoxia, oxidative stress, altered renal vasoreactivity, and volume depletion caused by osmotic diuresis [5]. Additionally, clinical conditions leading to stress hyperglycemia, such as sepsis or systemic inflammatory response, trauma or cardio-respiratory compromise may all contribute to and predict the propensity to develop AKI and death. Thus, hyperglycemia, irrespective to diabetes, may foresee the risk of AKI in patients presented with severe acute illness, as shown, for instance, in patients with COVID-19 disease [6]. Yet, while the impact of hyperglycemia upon admission on mortality has been thoroughly studied [7], data are limited regarding renal outcome [8,9,10], other than in the setup of acute myocardial infarction and coronary interventions [11,12,13,14]. Nevertheless, KDIGO guidelines suggest a correction of hyperglycemia among measures aimed at reducing AKI, for example in perioperative settings [15].

We have recently reported that the risk of AKI following radiocontrast-enhanced CT is generally negligible [16], with the exception of patients with advanced renal functional impairment [2], with diabetes being an independent predictor of contrast-induced nephropathy. It is noteworthy that a careful analysis of our database, which consisted of 41,456 inpatients undergoing CT, revealed a substantial number of patients displaying acute kidney functional recovery (AKR) following imaging, which was unrelated to the administration of contrast media [17]. In addition to a true recovery from renal injury, AKR among such patients, hospitalized with an acute illness, likely reflects the correction of fluid deficits, the restoration of hemodynamic stability and oxygenation, the amendment of metabolic derangements including hyperglycemia, the restoration of function of other organs, or the successful management of life-threatening infections. We hypothesized that this abrupt restoration of kidney function following imaging could mask the true incidence of contrast nephropathy. Additionally, since AKI and AKR in this cohort of patients closely correlated and were inversely associated with baseline kidney function, we further proposed that both could represent reduced renal functional reserve (RFR) [18].

A close association of AKI and AKR with pre-imaging glucose levels was found in this cohort of patients undergoing CT, supporting the masking effect of AKR in the assessment of contrast nephropathy among diabetics (Supplementary Table S1). As only a fraction of imaging procedures took place upon hospitalization (11.4%), we extended our study beyond patients undergoing CT, looking at the impact of blood glucose levels immediately upon admission on subsequent changes in renal function and mortality in all adult patients hospitalized in medical departments.

## 2. Materials and Methods

### 2.1. Study Design

This is a retrospective cohort analysis of electronic medical records of all inpatients admitted between 2012 and 2021 to a tertiary care facility in northern Israel, serving a population of 2.5 million people. The study was approved by the institutional review board at Rambam Health Care Campus (IRB RMB-D-0195-21).

### 2.2. Study Population and Data

All adult patients (≥18 years) that were admitted to the hospital and had available SCr and serum glucose taken in the Emergency Room or within three days before admission and at least one additional SCr sample taken between 24 h and 72 h after admission were included for analysis. For patients with repeated admissions, only the first one with available SCr and glucose values was included for analysis.

Data were obtained from a data extraction and synthetization platform [19]. Multiple patient baseline characteristics and parameters were extracted for analysis. These included demographics, medical diagnoses, vital signs, laboratory values, medications administered during the hospital stay, imaging studies, with or without contrast administration and interventional procedures such as angiography, endoscopy, or surgery. Medications were identified and classified by their Anatomical Therapeutic Chemical (ATC) classification. Surgery was defined as any surgical procedure. Contrast administration included all patients that underwent contrast-enhanced imaging or diagnostic/interventional angiography. Medical conditions were identified and grouped by their International Classification of Diseases (ICD9) codes. Admission time was considered as the index event, and for each variable, the value most proximal to the index event from 72 h before to 12 h after the index was considered as the baseline value for this variable. For continuous and categorical data, only variables that were available for at least 75% of patients and were positive in at least 5% of the patient population, respectively, were included. Only patients with available data for at least 75% of the remaining variables were included in the final analysis. For multivariable analysis, we input all missing information with median values for continuous variables and with the common value of each variable for dichotomous variables.

Estimated glomerular filtration rate (eGFR) was calculated using the chronic kidney disease (CKD) epidemiology collaboration formula [20].

### 2.3. Exposures and Outcomes

The main exposure variable was baseline serum glucose most proximal to the time of admission as defined above. Primary outcomes were AKI and AKR (acute renal functional recovery). AKI was diagnosed and staged when the difference between baseline serum creatinine (SCr) on admission and first SCr within 24 to 72 h after fulfilled the Kidney Disease Improving Global Outcomes (KDIGO) definition and staging system of AKI [21]. AKR was diagnosed as previously detailed [17], mirroring AKI definitions by KDIGO, when baseline SCr was higher by 0.3 mg/dL or was at least 1.5 times higher than subsequent SCr measurements obtained 24 to 72 h after admission. AKI and AKR stages 2–3 were defined with the SCr on admission being at least doubled on subsequent measurements 24–72 h later, or at least twice as high as subsequent measurements, respectively. Secondary outcome was all-cause mortality by 30 days from admission.

### 2.4. Statistical Analysis

All statistical analyses were performed using R version 4.0.3 (R Foundation for Statistical Computing). Patients were first divided to a low and high-glucose groups with a cutoff of 180 mg/dL in accordance with the target glucose range recommendation stated in the American Diabetes Association guidelines on diabetes management in hospitalized patients [22]. Baseline variables were compared between groups using the Mann–Whitney U-test and presented as medians with interquartile ranges for continuous variables and using the chi-square test and presented as absolute numbers and percentages for categorical variables.

To study the effect of baseline glucose on primary and secondary defined outcomes, we used a multivariable logistic regression model for each outcome variable. Patients with glucose levels in the lower or highest 0.5% percentile were excluded as outliers. In addition to baseline serum glucose levels, all variables that were found to be associated with renal outcomes were incorporated as covariates in the models. The impact of hyperglycemia on admission on covariables was determined per 100 mg/dL increment of glucose levels. Since hyperglycemia on admission in patients with diabetes may represent fluctuations in serum glucose associated with medical treatment, whereas hyperglycemia in non-diabetics is often a biomarker of stress response, the diagnosis of diabetes was added as a modifier to the effect of glucose. Similarly, since changes in renal function may affect glycosuria, eGFR was added as an additional modifier. We used cubic splines to plot the results and to extract glucose levels of the minimal and maximal adjusted probability of the categorical outcomes. The Hosmer–Lemeshow test was used to assess the model’s goodness of fit.

Additionally, we evaluated the correlation between the rates of AKI and AKR at varying eGFR values along the scale of 0 to 140 mL/min/1.73 m^2^, with observations grouped at intervals of 5 mL/min/1.73 m^2^.

## 3. Results

We analyzed 95,556 patients (52% male, mean age 61 years) that were admitted to the hospital with available SCr data for both specified baseline and 72 h follow-up values. A comparison of the baseline variables between patients with high serum glucose levels (>180 mg/dL, 16.5% of patients) and normal serum levels is presented in Table 1. Patients with higher glucose at presentation were older, with higher proportions of co-morbid conditions, including diabetes, had lower baseline renal function and were using more medications (Table 1). The rates of AKI, AKR, and mortality were almost doubled among patients with high baseline glucose levels (1552 (10%) vs. 4618 (6%) for AKI; 3849 (25%) vs. 9946 (13%) for AKR; and 1776 (11%) vs. 4499 (6%) for mortality, all with *p*-values < 0.001).

**Figure 1:**
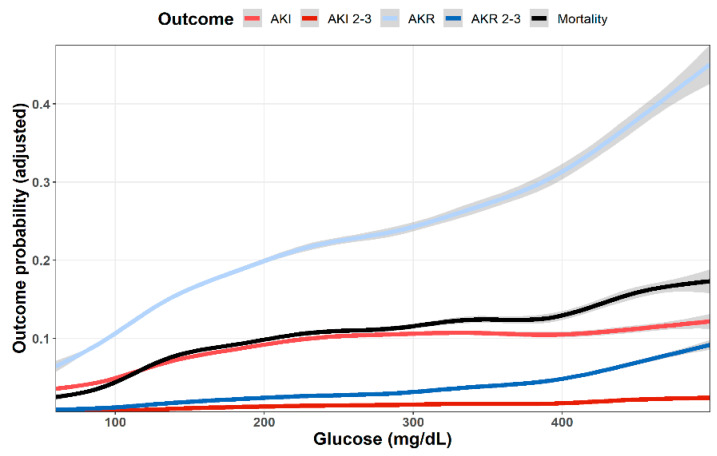
Predicted probability of various outcomes (AKI, AKI stages 2–3, AKR, AKR stages 2–3, dialysis and mortality at 30 days) as a function of baseline glucose levels. Lowest and highest 0.5% percentile of patients by glucose levels were excluded. Data were plotted using cubic splines.

**Figure 2:**
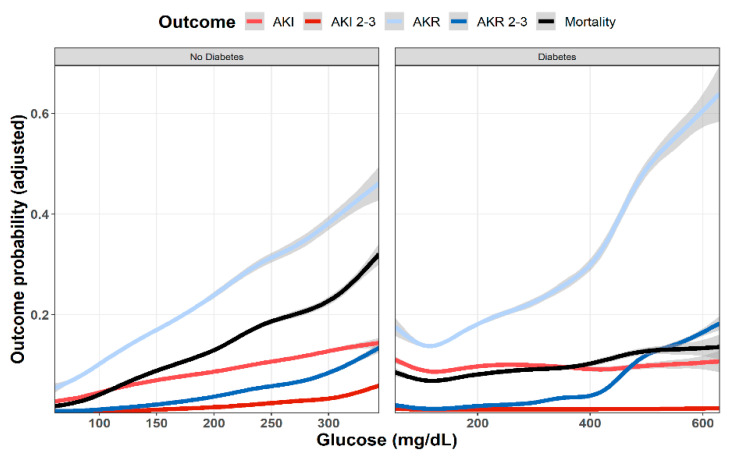
Predicted probability of various outcomes (AKI, AKR, and mortality at 30 days) as a function of baseline glucose levels in diabetic and non-diabetic patients. Lowest and highest 0.5% percentile of patients by glucose levels of each analyzed group were excluded. Data were plotted using cubic splines.

**Figure 3:**
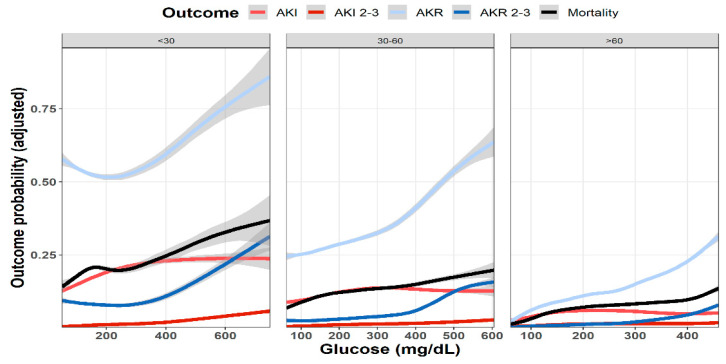
Predicted probability of various outcomes (AKI, AKR, and mortality at 30 days) as a function of baseline glucose levels in patients with preserved kidney function (eGFR > 60 mL/min/1.73 m^2^, *n* = 66,814), moderate renal dysfunction (60 > eGFR > 30 mL/min/1.73 m^2^, *n* = 20,822), and advanced renal failure (eGFR < 30 mL/min/1.73 m^2^, *n* = 8034). Lowest and highest 0.5% percentile of patients by glucose levels of each analyzed group were excluded. Data were plotted using cubic splines.

**Figure 4:**
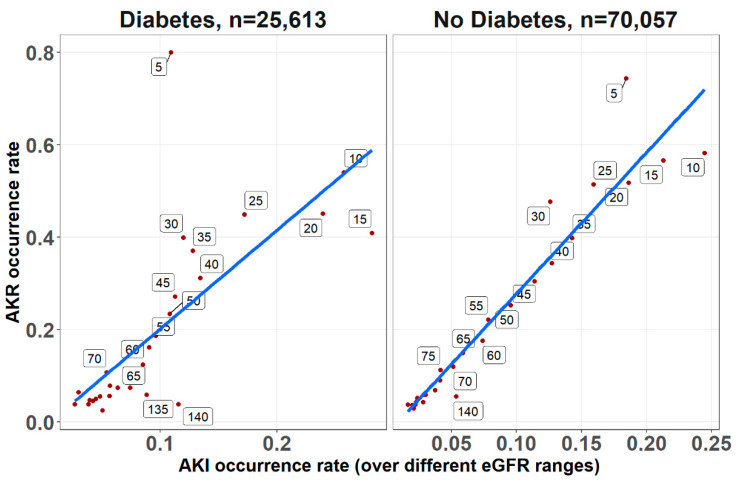
The correlations between AKI and AKR occurrence rates for diabetics and non-diabetic patients, determined across the range of baseline eGFR upon admission (at 5 mL/min/1.73 m^2^ intervals, denoted for each point along the regression lines), irrespective to glucose levels. Trend lines for the Pearson’s correlation coefficients are shown (r = 0.96 and r = 0.73 for non-diabetic and diabetic respectively, *p* < 0.001 for both).

As shown in Figure 1, multivariable analysis of the association of baseline glucose levels with renal and clinical outcomes demonstrated an increasing adjusted probability of all events as baseline glucose levels increased, with the most prominent rise seen in the rates of AKR. Figure 2 illustrates the association of baseline glucose levels with mortality and renal outcomes in diabetics and non-diabetic patients. Evidently, whereas the overall direct association shown in Figure 1 was also robust in non-diabetic patients, among diabetics, AKI and mortality were hardly affected by glucose levels upon admission. Figure 3 illustrates the association of baseline glucose levels with the same parameters, now with the patients stratified by their calculated baseline kidney function. Overall, the adjusted odds ratios of mortality, AKI, and AKR were inversely proportional to kidney function at baseline (i.e., increased odds ratios among patients with the lowest eGFR, Figure 3). Table 2 summarizes the adjusted odds ratios of AKI, AKR, and mortality, all increasing in proportion to baseline glucose levels. Notably, however, when looking at the interaction terms, diabetes was a modifier of the association of glucose and all outcomes with a substantial attenuating effect on the association of increase in glucose levels to the probability of outcomes (Table 2, Figure 2). Similarly, increasing eGFR at baseline was found to modify and enhance the effect of increasing glucose on the probability of AKR, whereas the effect on AKI has been moderated (Table 2, Figure 3). As shown in Figure 4, a highly significant correlation was noticed between AKI and AKR rates along the scale of baseline eGFR values. Plotted regression lines are illustrated separately for diabetic and non-diabetic patients (Pearson’s correlation coefficient r = 0.73 and r = 0.96 respectively, *p* < 0.001 for both plots).

## 4. Discussion

Our study confirms former reports, linking hyperglycemia with increased risk of AKI in acutely ill patients. As shown in Figure 1, the adjusted likelihood to develop AKI or AKI grades 2–3, as well as mortality were all proportional to the initial glucose levels upon admission. A similar association pattern was noted in non-diabetic patients, who were likely presenting with stress hyperglycemia (Figure 2). By contrast, hyperglycemia among diabetic patients was not associated with a significant rising risk of AKI or mortality. This group of patients likely included some with uncontrolled diabetes, but many patients might have had stress hyperglycemia on top, which was related to an acute illness.

When comparing patients grouped by their baseline kidney function, AKI and mortality increased in proportion to declining kidney function (Figure 3). Yet, whereas mortality was consistently associated with rising initial glucose levels, the impact of hyperglycemia on the adjusted likelihood to developed AKI leveled off roughly at glucose values of 200–300 mg/dL.

The adjusted odds ratios illustrated in Table 2 provides a statistical validation of these observations regarding glycemia–AKI association: whereas 100 mg/dL increments in glucose levels were associated with an increasing likelihood of AKI (OR 1.48, with 95% confidence intervals 1.33–1.65), this association was lost with interaction terms for both glucose–diabetes and glucose–eGFR co-associations.

Our observations analyzed by advanced statistical tools complement previous studies assessing glycemia–AKI association in acute clinical settings. Gordillo et al. [10] studying pediatric critically ill patients reported that AKI was associated with peak glycemia and that subjects on vasopressors had lower estimated glomerular filtration rate and higher glucose levels. Yet, glucose levels were not associated with urine or plasma levels of neutrophil gelatinase-associated lipocalin (NGAL), which is a biomarker of injury at distal tubular segments. By contrast, Wang et al. [8] reported that among non-diabetic patients with stress hyperglycemia and AKI, glucose levels correlated with urinary N-acetyl-β-D-glucosaminidase (uNAG), which is another marker of tubular injury. Guvercin et al. [9] found that serum matrix metalloproteinase (MMP)-9 levels were associated with the need for dialysis and with mortality among non-diabetic geriatric patients with stress hyperglycemia and AKI. Moriyama et al. reported that hyperglycemia upon admission was found to be an independent predictor of AKI among 664 Japanese patients presented with acute myocardial infarction (AMI), a third of them, only, with diabetes [14]. Shacham et al. further studied the association of glucose levels upon admission with the risk of AKI among 1061 non-diabetic patients with ST-segment elevation myocardial infarction (STEMI). They found that severe hyperglycemia emerged as an independent predictor of AKI, with an odds ratio of 2.46 [13]. The association of hyperglycemia and AKI was also studied selectively among diabetics in a similar setup of acute myocardial infarction (AMI). In this population, glucose levels upon admission could reflect both overall glycemic control and the impact of stress. Therefore, stress hyperglycemia ratio has been designed and applied, looking at current glucose levels relative to chronic glycemia, which were extrapolated from glycated hemoglobin values. Marenzi et al. studied 474 diabetic patients with AMI and found that the incidence of AKI increased in parallel with the acute/chronic glycemic ratio but not with admission glycemic tertiles [12]. Likewise, Gao et al. evaluated 1215 diabetic patients with AMI and found that AKI and mortality were associated with stress hyperglycemia ratio upon admission [11]. Collectively, these studies are in line with our findings, indicating that stress hyperglycemia at presentation, rather than non-stress hyperglycemia in diabetics, predicts AKI in acutely ill inpatients, whereas the later association could simply reflect altered adherence to medical control of diabetes prior to admission.

AKR among inpatients with hyperglycemia, a mirror image of AKI reflects renal functional recovery that could result from diverse causes. Correction of fluid losses and circulatory failure is detrimental for volume depletion related to osmotic diuresis. Managing sepsis and the restoration of metabolic derangements and function of other organs are among common additional mechanisms involved in the AKR phenomenon, as is withholding medications that alter glomerular hemodynamics, such as inhibitors of the rennin–angiotensin axis or of sodium–glucose co-transport (SGLT2 inhibitors). AKR may also represent the recovery from tubular injury induced by the acute illness and its complications. On the other hand, AKR might conceal the occurrence of in-hospital iatrogenic and other renal insults with subclinical AKI that may be detected clinically only by biomarkers of tubular injury [23].

Evaluating patients undergoing enhanced and non-enhanced CT, we have previously reported that the likelihood of developing AKR was closely associated with that of AKI along the scale of baseline kidney function, and that both AKI and AKR were directly proportional to renal functional impairment [18]. As illustrated in Figure 3, AKI and AKR probabilities were higher as baseline eGFR declined, which is in line with our previous observations among inpatients following CT imaging [17]. Furthermore, AKI and AKR were co-associated (Figure 4) and inversely correlated with baseline eGFR (i.e., higher probabilities at lower eGFR, as illustrated in Table 2 and Figure 3). These findings, now in a much larger cohort of patients, in a broader clinical setting irrespective to imaging, consolidates our hypothesis that both AKI and AKR reflect a shared pathophysiology, namely a loss of renal functional reserve, as recently discussed in detail elsewhere [18].

As shown in Figure 1, the curves showing adjusted probability of AKI and AKR were concordant along the scale of glycemia, both increasing with rising glucose levels. Yet, the adjusted probability of AKR was substantially higher than that of AKI, especially at the higher range of glucose levels. Moreover, whereas AKI probability leveled at glucose concentration of 250 mg/dL, that of AKR steeply increased further and was three to four times higher than AKI as glucose levels exceeded 400 mg/dL (Figure 1). This dichotomy at higher glucose levels suggests additional physiologic components. Figure 2 provides some insight for that phenomenon, clearly showing that whereas in non-diabetics with stress hyperglycemia, increasing AKR probability remains proportional to rising glucose concentrations (as do AKI and mortality), among diabetics, AKR likelihood, only, keeps rising. Since the majority of hyperglycemic patients were diabetics, this affects the overall predominance of AKR over AKI illustrated in Figure 1. We propose that the different pat-terns of AKI/AKR association in diabetics vs. non diabetics reflect additional factors affecting AKR but not AKI, which may not be related to reversible tubular injury, such as the rapid restoration of volume depletion provoked by osmotic diuresis, withholding RAAS blockers or SGLT2 inhibitors during the acute illness, or the resumption of interrupted medications controlling glucose levels.

As shown in Supplementary Table S1, non-diabetic patients with stress hyperglycemia were much sicker than diabetics, with a higher mortality rate, and that likely explains the rising probability to develop AKI at higher glucose levels, which presumably reflects the degree of critical disease scores [24]. Parallel dichotomy regarding the effect of hyperglycemia upon outcome between patients with stress hyperglycemia and diabetics without stress hyperglycemia has indeed been reported in other clinical scenarios, such as cerebrovascular events [25] and acute myocardial infarction [26], underscoring the important role of stress response and injury of other organs in the induction of AKI.

Notably, our data analysis findings, addressing plasma glucose upon admission (Table 2), largely parallel those found in the smaller cohort of patients undergoing CT imaging throughout the hospitalization course (only 11.4% of them performed upon admission), as illustrated in Supplementary Table S2. In both cohorts, while pre-imaging high-glucose concentrations predicted increased likelihood to develop AKI or AKR, or to die, the glucose–diabetes interaction resulted in a reduced likelihood prediction of their development, underscoring the predominant impact of disease severity and stress hyperglycemia. The same can be said regarding the interaction terms of glucose and eGFR (Table 2), with reversal (AKI) or attenuation (AKR and mortality) of the effect of hyperglycemia on the adjusted outcome probabilities.

Patients who were discharged from the hospital or died before an additional SCr was taken at the prespecified interval were not included in the study, which is a possible source of selection bias. However, the study population was selected to address the principal aim of this study, which was to assess the association between blood glucose levels upon admission and renal outcomes in hospitalized patients, while the association of blood glucose and various other outcomes was thoroughly reported [7].

The strengths of this report are its large size, and the detailed available information per patient during the hospitalization course. It also provides for the first time an overall perspective and insight regarding changing renal function in hospitalized patients encompassing an in-depth evaluation of AKI, AKR, and their associations. Our study also suffers a few limitations, first—being a single-center study. This drawback is partially compensated for by the inclusion of patients from all medical wards where medical teams operate independently. Yet, it does not include patients of all races, pediatric patients, or those hospitalized in surgical and obstetric wards. Out-of-hospital medical recordings, including medications at home, are likely incomplete and may be inaccurate. Most importantly, since HbA1C levels are not routinely determined among hospitalized patients in Rambam Health Care Campus, we were unable to specifically define the stress hyperglycemia ratio in our patients, as performed in other relevant studies [11,12], precluding the clear-cut determination of stress hyperglycemia related to an acute illness in our diabetic patients. Consequently, the group of diabetic patients shown in Figure 2 likely represents a mixture of patients with and without stress hyperglycemia. There is also the obvious limitation of using plasma creatinine as an indicator of changing renal function under unsteady conditions. Lastly, since patients with glucose levels in the lower or highest 0.5% percentile were excluded as outliers for statistical robustness, we likely ignored some patients with diabetic emergencies such as non-ketotic hyperosmolar state or diabetic ketoacidosis.

In conclusion, stress hyperglycemia is associated with AKI, AKR, and mortality in non-diabetic inpatients, likely reflecting disease severity. Yet, this association is significantly diminished in diabetic patients, suggesting a possible impact of treatable and easily reversible renal derangement in this population during the management of an acute illness. AKR accompanies convalescence to a much larger extent than AKI among inpatients, likely reflecting the successful management of acute systemic deterioration that affects kidney function. AKR and AKI are closely associated and are inversely related to kidney function. The AKR phenomenon following hospital admission possibly undermines the assessment of subclinical AKI caused by additional in-hospital renal insults, and it plausibly reflects reduced RFR.

## Figures and Tables

**Table 1:** Comparison of baseline variables, renal outcomes, and mortality between patients with high baseline serum glucose (>180 mg/dL) and lower-to-normal baseline serum glucose (≤180 mg/dL). Continuous variables are presented as medians with interquartile ranges. Categorical variables are presented as absolute numbers and percentages.

Variable	High Glucose (*n* = 15,675)	Low Glucose (*n* = 79,555)	*p*-Value	Total (*n* = 95,755)
Age, years	69.7 (20.2)	62.2 (32.9)	<0.001	63.9 (30.6)
Male, *n* (%)	8442 (54%)	40,931 (51%)	<0.001	49,373 (52%)
Diagnosis, *n* (%)				
Diabetes	11,262 (72%)	14,266 (18%)	<0.001	25,528 (27%)
Hyperlipidemia				
Hypertension	2071 (13%)	5592 (7%)	<0.001	7663 (8%)
IHD	4026 (26%)	11,772 (15%)	<0.001	15,798 (17%)
Heart failure	1479 (9%)	3490 (4%)	<0.001	4969 (5%)
COPD	1246 (8%)	4378 (6%)	<0.001	5624 (6%)
Vital signs				
Heart rate, bpm	88 (26)	83 (24)	<0.001	84 (25)
Systolic blood pressure, mmHg	143 (42)	135 (34)	<0.001	136 (35)
Temperature, C	36.8 (0.5)	36.8 (0.4)	<0.001	36.8 (0.4)
Oxygen saturation, %	96 (4)	97 (4)	<0.001	97 (4)
Laboratory data				
Hemoglobin, g/dL	12.4 (3)	12.7 (2.8)	<0.001	12.7 (2.8)
White blood cells, k/uL	11.2 (6.9)	9.6 (5.3)	<0.001	9.8 (5.7)
Platelets, k/uL	230 (113)	222 (103)	<0.001	223 (105)
Creatinine, mg/dL	1.1 (0.7)	0.9 (0.4)	<0.001	0.9 (0.5)
Blood urea nitrogen, mg/dL	21 (17)	16 (10.5)	<0.001	16.6 (12)
Estimated glomerular filtration ratio, mL/min/1.73 m^2^	60.3 (44.4)	83.6 (45.2)	<0.001	80.1 (46.8)
Medications and interventions, *n* (%)				
Loop diuretics	4757 (30%)	12,688 (16%)	<0.001	17,445 (18%)
Renin–angiotensin–aldosterone system inhibitors	7400 (47%)	24,284 (31%)	<0.001	31,684 (33%)
Insulin	7415 (47%)	4599 (6%)	<0.001	12,014 (13%)
Metformin	4725 (30%)	6589 (8%)	<0.001	11,314 (12%)
Fluids	9491 (61%)	44,163 (56%)	<0.001	53,654 (56%)
Surgery	1989 (13%)	11,638 (15%)	<0.001	13,627 (14%)
Contrast administration	1974 (13%)	8944 (11%)	<0.001	10,918 (11%)
Outcomes, *n* (%)				
AKI	1552 (10)	4618 (6)	<0.001	6170 (6)
AKI 2–3	233 (2)	639 (1)	<0.001	872 (1)
AKR	3849 (25)	9946 (6)	<0.001	13,975(14)
AKR 2–3	576 (4)	1129 (1)	<0.001	1705 (2)
Mortality	1776 (11)	4499 (6)	<0.001	6275 (7)

IHD, ischemic heart disease; COPD, chronic obstructive pulmonary disease; AKI, acute kidney injury; AKR, acute kidney recovery.

**Table 2:** Adjusted odds ratios of variables and interaction terms for the logistic regression models of acute kidney injury, acute kidney recovery and mortality.

	Adjusted Odds Ratio (95% Confidence Interval)		
	AKI	AKR	Mortality
Glucose (per increase of 100 mg/dL)	1.48 (1.33, 1.65)	1.27 (1.17, 1.38)	1.76 (1.59, 1.95)
Secondary effects			
Age (per increase of 1 year)	1.01 (1.01, 1.01)	0.97 (0.97, 0.97)	1.05 (1.04, 1.05)
Blood urea nitrogen (per increase of 1 mg/dL)	1.00 (1.00, 1.10)	1.01 (1.01, 1.01)	1.02 (1.02, 1.02)
Diabetes	1.40 (1.20, 1.63)	1.20 (1.06, 1.35)	1.55 (1.33, 1.81)
Heart failure	1.18 (1.07, 1.29)	0.82 (0.75, 0.90)	0.81 (0.72, 0.91)
Hypertension	1.01 (0.93, 1.10)	1.11 (1.03, 1.20)	1.00 (0.92, 1.10)
Ischemic heart disease	1.05 (0.97, 1.12)	0.86 (0.81, 0.91)	0.78 (0.72, 0.84)
Systolic blood pressure (per increase of 10 mmHg)	1.04 (1.03, 1.05)	0.89 (0.89, 0.90)	0.92 (0.91, 0.93)
Temperature (per increase of 1 C)	0.92 (0.88, 0.95)	1.21 (1.17, 1.25)	0.92 (0.89, 0.96)
Hemoglobin (per increase of 1 g/dL)	0.96 (0.95, 0.98)	1.08 (1.07, 1.09)	0.92 (0.91, 0.93)
eGFR (per increase of 30 mL/min/1.73 m^2^)	0.87 (0.81, 0.93)	0.19 (0.18, 0.20)	1.10 (1.02, 1.18)
Insulin	1.45 (1.33, 1.57)	0.71 (0.66, 0.77)	1.30 (1.19, 1.42)
RAAS inhibitors	0.81 (0.76, 0.86)	0.87 (0.83, 0.92)	0.53 (0.50, 0.57)
Loops diuretics	2.43 (2.28, 2.58)	0.56 (0.53, 0.6)	1.77 (1.66, 1.89)
Fluids	1.32 (1.25, 1.40)	1.84 (1.76, 1.93)	2.22 (2.08, 2.38)
Contrast administration	1.08 (0.99, 1.18)	1.58 (1.48, 1.68)	1.54 (1.42, 1.68)
Surgery	1.58 (1.47, 1.70)	1.13 (1.06, 1.21)	0.61 (0.56, 0.68)
Interaction Terms			
Diabetes–glucose	0.78 (0.72, 0.86)	0.81 (0.76, 0.87)	0.62 (0.57, 0.67)
eGFR–glucose	0.88 (0.85, 0.92)	1.20 (1.16, 1.24)	1.04 (1.00, 1.08)

AKI, acute kidney injury; AKR, acute kidney recovery; eGFR, estimated glomerular filtration ratio; RAAS, renin–angiotensin–aldosterone system.

## Data Availability

Data sharing requires institutional authorization. For additional information, please contact corresponding author.

## Supplementary Materials

The following are available online at https://www.mdpi.com/article/10.3390/jcm11010054/s1, Table S1: Comparison of baseline variables, renal outcomes, and mortality in hospitalized patients with high baseline serum glucose (>180 mg/dL). Compared are patients with or without diabetes; Table S2: Prediction of renal outcome and mortality among inpatients undergoing contrast-enhanced (*n* = 10,026) or non-enhanced computerized tomography (*n* = 27,451): Shown are ORs with 95% CIs results following multivariate logistic regression including pre-imaging glucose levels, diabetes and other covariates.
