# Supplementary material for: Hyperglycemia on Admission Predicts Acute Kidney Failure and Renal Functional Recovery among Inpatients
Source: J Clin Med. 2021 Dec 23;11(1):54. doi: 10.3390/jcm11010054 (PMC8745405; doi:10.3390/jcm11010054)
Supplement: Supplementary file 1 [file jcm-11-00054-s001.zip › jcm-1485222-supplementary.pdf]

Table S1. Comparison of baseline variables, renal outcomes, and mortality in hospitalized patients with high baseline serum glucose (>180 mg/dL). Compared are patients with or without diabetes. .

Variable	With diabetes (n=11,262)	No diabetes (n=4,113)	P-value	Total (n=15,675)
Age, years	70 (18.4)	68.9 (26.1)	<0.001	69.7 (20.2)
Male, n (%)	5,991 (53%)	2,451 (56%)	<0.001	8,442 (54%)
**Diagnoses, n (%)**				
Hypertension	1,787 (16%)	284 (6%)	<0.001	2,071 (13%)
IHD	3,361 (30%)	665 (15%)	<0.001	4,026 (26%)
Heart failure	1,193 (11%)	286 (6%)	<0.001	1,479 (9%)
COPD	897 (8%)	349 (8%)	0.93	1,246 (8%)
**Vital signs**				
Heart rate, bpm	88 (24)	90 (30)	0.001>	88 (26)
Systolic blood pressure, mmHg	145 (43)	137 (39)	0.001>	143 (42)
**Temperature, ^{0}C**	36.8 (0.5)	36.8 (0.6)	0.001>	36.8 (0.5)
Oxygen saturation, %	96 (4)	97 (5)	0.001>	96 (4)
**Laboratory data**			0.001>	
Hemoglobin, g/dL	10.7 (3.4)	10.3 (3.7)	0.001>	10.6 (3.4)
White blood cells, k/ul	10.6 (6.2)	13 (8.3)	0.001>	11.2 (6.9)
Platelets, k/ul	195 (105)	172 (105)	0.001>	188 (106)
Creatinine, mg/dL	1.1 (0.7)	1.1 (0.6)	0.001>	1.1 (0.7)
Blood urea nitrogen, mg/dL	22 (17.6)	19 (13.8)	0.001>	21 (17)
Estimated glomerular filtration ratio, ml/min/m²	58.3 (44.7)	65.8 (43.4)	0.001>	60.3 (44.4)
**(%) Medications and interventions, n**				
Loop diuretics	3648 (32%)	1109 (25%)	0.001>	4757 (30%)
Renin-angiotensin-aldosterone system inhibitors	6074 (54%)	1326 (30%)	0.001>	7400 (47%)
Fluids	6678 (59%)	2813 (64%)	0.001>	9491 (61%)
Surgery	1124 (10%)	865 (20%)	0.001>	1989 (13%)
Contrast administration	1134 (10%)	840 (19%)	0.001>	1974 (13%)
**Outcomes, n (%)**				
AKI	1,106 (10%)	446 (10%)	0.61	1,552 (10%)
AKI2	131 (1%)	102 (2%)	0.001>	233 (1%)
AKR	2,628 (23%)	1,221 (28%)	0.001>	3,849 (25%)
AKR2	357 (3%)	219 (5%)	0.001>	576 (4%)
Mortality	1,041 (9%)	735 (17%)	0.001>	1,776 (11%)

IHD, ischemic heart disease; COPD, chronic obstructive pulmonary disease; AKI, acute kidney injury; AKR, acute kidney recovery.

Table S2. Prediction of renal outcome and mortality among inpatients undergoing contrast-enhanced (n=10,026) or non-enhanced computerized tomography (n=27,451): Shown are ORs with 95% CIs results following multivariate logistic regression including pre-imaging glucose levels, diabetes and other covariates.

Outcome with OR (95% CI)				
**Mortality**	**Dialysis**	**AKR**	**AKI**	
1.82 (1.66, 1.99)	1.69 (1.32, 2.11)	2.14 (1.98, 2.32)	1.55 (1.39, 1.72)	**Glucose (OR per 100 mg/dL increase)**
0.63 (0.57, 0.71)	0.61 (0.47, 0.81)	0.66 (0.6, 0.72)	0.65 (0.58, 0.74)	**Glucose-Diabetes interaction**
1.04 (1.04, 1.04)	1 (0.99, 1)	1 (1, 1.01)	1.01 (1.01, 1.02)	Age (OR per 1 year increase)
1.06 (0.97, 1.15)	0.94 (0.73, 1.2)	0.99 (0.92, 1.07)	1.03 (0.93, 1.13)	Contrast-enhanced imaging
				**Chronic diseases**
1.62 (1.35, 1.95)	2.91 (1.84, 4.56)	1.57 (1.33, 1.85)	2.07 (1.69, 2.53)	Diabetes
0.72 (0.63, 0.83)	2.6 (2.03, 3.33)	0.5 (0.44, 0.57)	1.67 (1.47, 1.88)	Chronic kidney disease
0.84 (0.77, 0.92)	0.82 (0.63, 1.08)	1.09 (1.01, 1.19)	1.13 (1.01, 1.26)	Hypertension
0.99 (0.91, 1.08)	0.98 (0.77, 1.23)	1.04 (0.96, 1.13)	1 (0.9, 1.1)	Ischemic heart disease
1.06 (0.96, 1.17)	1.4 (1.1, 1.78)	0.9 (0.81, 0.99)	1.32 (1.18, 1.47)	Heart Failure
1.2 (1.09, 1.31)	0.94 (0.73, 1.2)	0.85 (0.78, 0.93)	0.96 (0.86, 1.06)	Cerebrovascular disease
				**Vital signs**
0.99 (0.99, 0.99)	1 (0.99, 1)	0.99 (0.99, 0.99)	1 (1, 1)	SBP (OR per 1 mmHg increase)
				**Laboratory results**
1.21 (1.18, 1.25)	1.64 (1.58, 1.71)	2.31 (2.22, 2.4)	1.21 (1.18, 1.24)	Creatinine (OR per 1 mg/dL increase)
0.89 (0.87, 0.9)	0.87 (0.84, 0.9)	1.06 (1.04, 1.07)	0.95 (0.94, 0.97)	Hb (OR per 1 g/dL increase)
1.02 (1.01, 1.02)	1 (1, 1.01)	1.01 (1.01, 1.02)	1.01 (1, 1.01)	WBC (OR per 1 ul increase)
				**Medical treatment**
0.81 (0.72, 0.9)	0.94 (0.69, 1.24)	0.64 (0.58, 0.71)	1.05 (0.94, 1.17)	RAAS inhibitors
1.27 (1.18, 1.38)	1.38 (1.13, 1.7)	1.41 (1.32, 1.51)	1.07 (0.98, 1.17)	Antibiotics
1.85 (1.65, 2.08)	1.76 (1.35, 2.28)	0.62 (0.54, 0.71)	1.72 (1.52, 1.95)	Loop diuretics IV
0.92 (0.82, 1.03)	1.14 (0.86, 1.49)	1.05 (0.95, 1.15)	0.84 (0.73, 0.96)	Glucose-containing fluids

AKI, acute kidney injury; AKR, acute kidney recovery; eGFR, estimated glomerular filtration ratio; RAAS, renin-angiotensin-aldosterone system; SBP, systolic blood pressure; Hb, hemoglobin; WBC, white blood cells.; IV, intravenous;
